# Maus’s salts: an old family of iron sulfates with possible magnetic frustration

**DOI:** 10.1107/S2052520626002027

**Published:** 2026-05-07

**Authors:** Analeece Long, Matthew Powell, Ashley Dickey, Colin McMillen, Joseph Kolis

**Affiliations:** a Department of Chemistry Clemson University Clemson SC29634-0973 USA; Siberian Branch of Russian Academy of Science, Russian Federation

**Keywords:** Maus’s salt, magnetic frustration, crystal structure, crystal growth

## Abstract

The family of Maus’s salts has been known for several centuries, but many aspects of their structures remain elusive, particularly because most examples are highly solvated and readily lose water. To examine the role of alkali cations and water on the structure, and to resolve some ambiguities of previously reported supercells, several new Maus’s salt derivatives were synthesized and characterized by low-temperature X-ray diffraction. The parent Maus’s salt, K_5_[Fe_3_O(SO_4_)_6_(H_2_O)_3_]·5H_2_O, grows in hexagonal *P*6_3_/*m*, with trigonal symmetry of the Fe^3+^ clusters, conditions that allow for potential magnetic frustration. A new caesium ferric sulfate complex, Cs_5_[Fe_4_O_2_(HSO_4_)(SO_4_)_6_(H_2_O)_3_]·1.75H_2_O, was also synthesized under the same conditions. It is not a Maus’s salt derivative but instead contains Fe^3+^ tetramers of corner- and edge-sharing iron octahedra, decorated by seven sulfate tetrahedra, crystallizing in monoclinic space group *C*2/*c*. Preliminary orientation-dependent magnetic data relative to the trigonal *c* axis were collected on single crystals of the parent Maus’s salt. The data suggests very weak short-range antiferromagnetic spin reorientations with a subtle broad rolling feature having a local maximum near 60–70 K. Magneto-structural anisotropy and spin saturation moments, one-tenth those expected for high-spin Fe^3+^, suggest a splayed magnetic structure with spin moments predominantly in the trimer plane directed inward about the local *z* axis toward the shared oxygen between trimers.

## Abstract 2

Structural investigations into Maus’s salt derivatives reveal great sensitivity toward alkali metal and cocrystallized water content. Magnetic properties of the Maus’s salt iron trimer are explored.

## 1. Introduction

Of the extensive array of iron sulfates known (Alpers *et al.*, 2018 ▸; Schindler *et al.*, 2006 ▸; Hawthorne *et al.*, 2000 ▸), one of the oldest is a relatively obscure class known as the Maus’s salt derivatives. Maus’s salt represents one of the first rational syntheses of any iron-based compound, having first been reported by Maus (1827 ▸) and subsequently reinvestigated in a short but remarkable paper by Haidinger (1854 ▸). He succeeded in describing a rational high-yield synthesis of the material, growing large, high-quality hexagonal single crystals, proposing a reasonable formulaic ratio, identifying waters of hydration in the formula and observing its dichroic nature, all of which subsequently proved to be essentially correct. This compound turned out to be merely the first in wide range of related derivatives.

**Figure 1:**
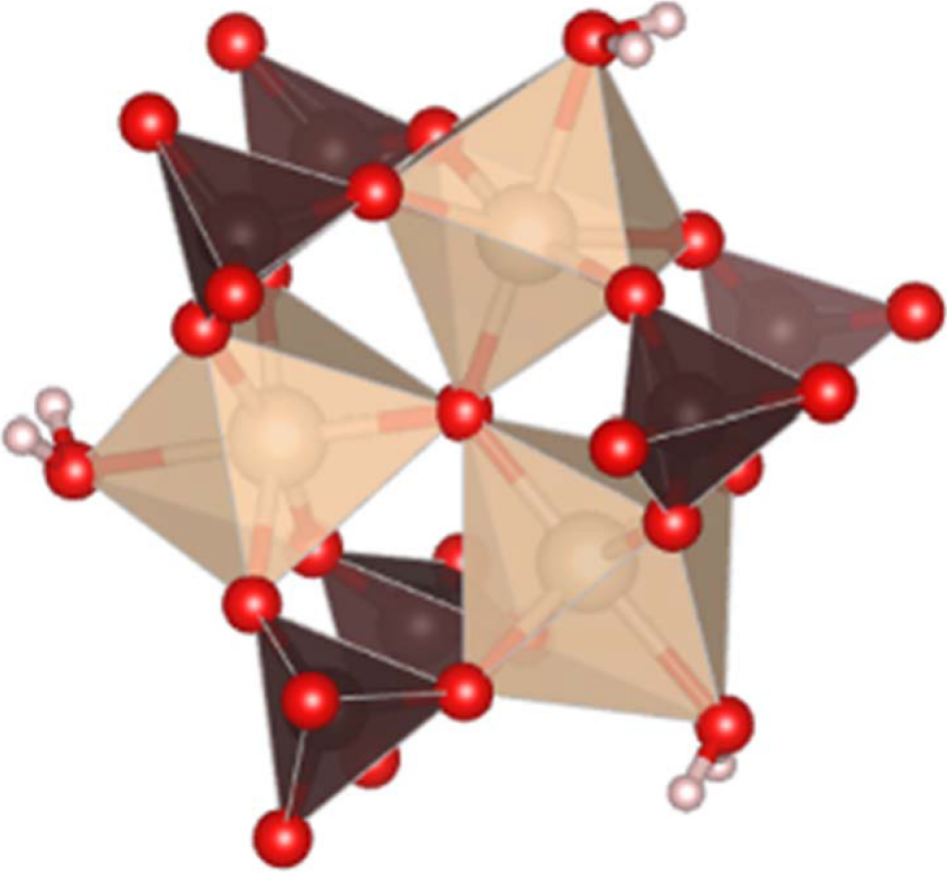
Maus’s salt trimer building block. Iron atoms are shown as tan octahedra, sulfur atoms are shown as brown tetrahedra, oxygen atoms are shown as red spheres, and hydrogen atoms are shown as white spheres.

The large number of Maus’s salts are all derived from the parent compound K_5_[Fe_3_O(SO_4_)_6_(H_2_O)_3_]·5H_2_O, which contains a triangular building block containing Fe^3+^ ions linked by sulfate groups and a central oxygen. Each Fe^3+^ ion also contains a coordinated water, and these ligands all combine to generate an approximately octahedral coordination environment around each Fe^3+^ center. These penta-anionic ferric trimers, [Fe_3_O(SO_4_)_6_(H_2_O)_3_]^5−^, constitute the primary building block of all the Maus’s salts (Fig. 1 ▸). Beyond this building block, however, there is considerable structural ambiguity. This includes the identity of cationic species as well as the amount of co-crystallized water present in the regions between trimers (Giacovazzo *et al.*, 1975 ▸).

A critical variable in Maus’s salt derivatives is the role of the alkali metal ion and its associated coordinating water molecules. Among the various known derivatives, the most common alkali ion is potassium but various other combinations of sodium, rubidium and even ammonium have been reported (Mereiter & Völlenkle, 1978 ▸; Mereiter & Völlenkle, 1980 ▸; Mereiter, 1990 ▸; Scordari & Stasi, 1990 ▸; Mereiter, 2013 ▸). Typically, the ferric trimers are arrayed in planes with the hydrated alkali ions located between the planes generating complex quasi two-dimensional structures. While the parent Maus’s salt adopts hexagonal symmetry, other members of the family are crystallographically trigonal or have lower (typically monoclinic) symmetry even though the trigonal ferric building blocks remain intact. The reduction in symmetry is often not extreme and is usually only the result of relatively minor movements of the trimers within the layers. Thus, in many cases the structures contain two axes with similar distances (*ca* 9.5 Å) while the third axis (normal to the layers of trimers) varies widely because of the enormous variability of the stacking options depending on the amount of interlayer water molecules and their hydrogen bonding arrangements.

The complex chemistry of Maus’s salt derivatives is often the result of water molecules not directly coordinated to the ferric ions. These water molecules have several functions, many of which rely on hydrogen bonding, either with coordinated sulfates, or other water molecules coordinated to alkali ions or even exist as lone waters of hydration within the lattice. These water molecules are highly labile and often readily dehydrate, adding to instability of the compounds and problems with accurate structural solution. Most of the structures are extremely sensitive to even minor variations in growth conditions and often the slightest difference in alkali ion identity, pH, stoichiometry, growth temperature, and other factors lead to different structures and formulas. In this paper, we report a series of low-temperature X-ray diffraction data resulting in accurate solution of a number of Maus’s salts, including that of the parent compound for the first time. We also report a new related Cs^+^-containing compound that is not a Maus-type structure, as well as the first magnetic study of the parent compound, which displays elements of trigonal magnetic frustration.

## 2. Experimental

### 2.1. Synthesis of Maus’s salt derivatives

**Figure 2:**
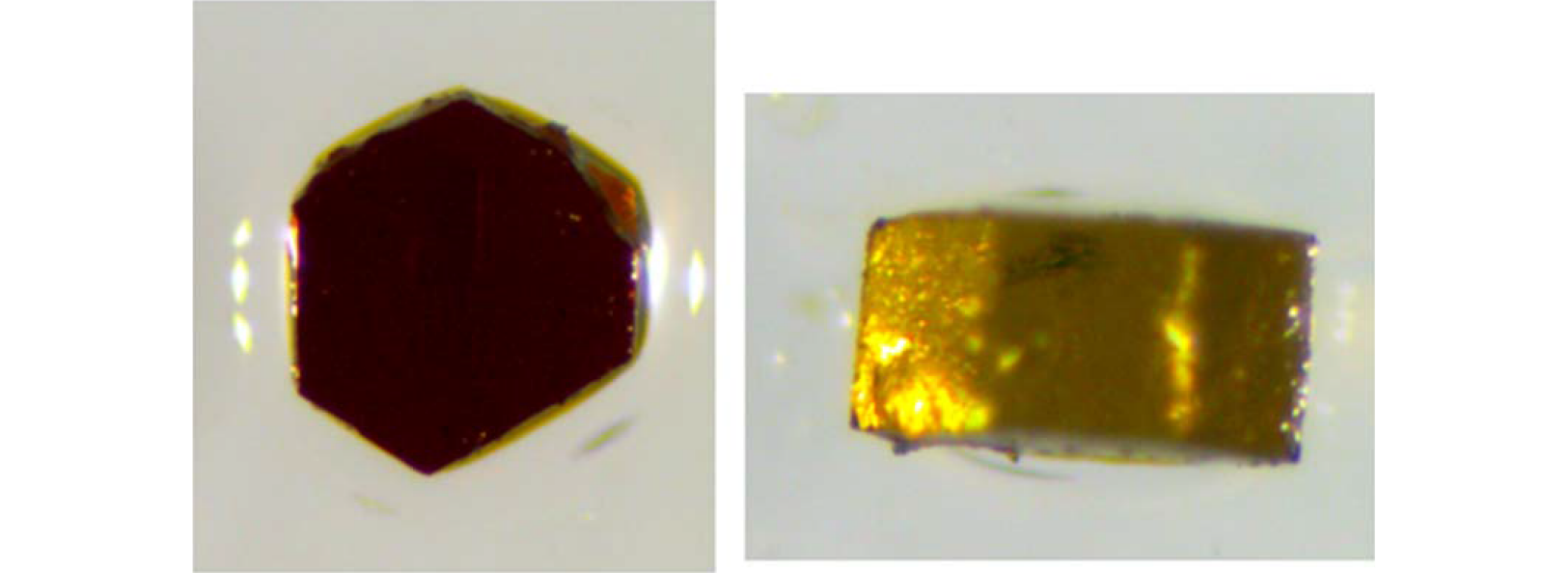
Typical well formed 2 mm-diameter by 1 mm-thick single crystals of Maus’s salt.

Large, millimetre-sized single crystals were prepared through general evaporative solution methods in 20 ml scintillation vials. Crystal growth reactions were performed in aqueous solutions of an alkali sulfate [Rb_2_SO_4_ (99.8%, Thermo-Fisher); K_2_SO_4_ (>99%, BTC); Cs_2_SO_4_ (99.9%, Chem-Implex); Na_2_SO_4_ (>99%, ACS)] and Fe_2_(SO_4_)_3_·5H_2_O (97%, Acros) as noted in subsections below. Each solution was gently heated and stirred to ensure a homogeneous mixture, then left to evaporate with or without perforated covering. The resulting brown hexagonal crystals (Fig. 2 ▸) were collected and found together with the corresponding alkali-alum. The crystals experienced different degrees of deterioration when exposed to atmospheric conditions, thus they were retained in the mother liquor or covered in mineral oil until further use.

#### 2.1.1. K_5_[Fe_3_O(SO_4_)_6_(H_2_O)_3_]·5H_2_O (**I**)

A 1:2 molar ratio of K_2_SO_4_ (0.343 g, 1.97 mmol) and Fe_2_(SO_4_)_3_·5H_2_O (1.94 g, 3.95 mmol) was dissolved in deionized water (10 ml) followed by slow evaporation to produce crystals.

#### 2.1.2. Rb_5_[Fe_3_O(SO_4_)_6_(H_2_O)_3_]·5H_2_O (trigonal) (**II**)

A 1:2 molar ratio of Rb_2_SO_4_ (0.500 g, 2.72 mmol) and Fe_2_(SO_4_)_3_·5H_2_O (0.660 g, 1.35 mmol) was added to deionized water (5 ml). This combination was evenly heated at 100°C to obtain a homogeneous solution which was allowed to evaporate to produce crystals.

#### 2.1.3. Rb_5_[Fe_3_O(SO_4_)_6_(H_2_O)_3_]·2H_2_O (monoclinic) (**III**)

A 1:2 molar ratio of Rb_2_SO_4_ (0.500 g, 2.72 mmol) and Fe_2_(SO_4_)_3_·5H_2_O (0.660 g, 1.35 mmol) was added to deionized water (5 ml). Following light stirring, a homogeneous orange solution was obtained. A NaOH solution (10 *M*, 0.075 ml) was added dropwise, with continuous heating at 90°C, and stirred overnight until crystal formation was observed. The vial was then removed from the heat when 1–2 ml solution remained to prevent complete dehydration.

#### 2.1.4. (Na_1.8_Rb_3.2_)[Fe_3_O(H_2_O)_3_(SO_4_)_6_]·5H_2_O (**IV**)

A 1:1:1 molar ratio of Na_2_SO_4_ (0.280 g, 1.97 mmol), Rb_2_SO_4_ (0.526 g, 1.97 mmol) and Fe_2_(SO_4_)_3_ (0.788 g, 1.97 mmol) was added to deionized water (10 ml). The solution was stirred and warmed gently, then removed from heat and left to evaporate to produce crystals.

#### 2.1.5. Cs_5_[Fe_4_O_2_(HSO_4_)(SO_4_)_6_(H_2_O)_3_]·1.75H_2_O (**V**)

A 1:2 molar ratio of Cs_2_SO_4_ (0.116 g, 1.97 mmol) and FeSO_4_ (0.600 g, 3.94 mmol) was added to deionized water (5 ml) and methanol (5 ml). The solution was gently heated and stirred, followed by gravity filtration to remove undissolved solids. The filtrate solution was left in a parafilm-covered vial with small holes to allow slow evaporation under ambient conditions.

### 2.2. Single-crystal X-ray diffraction

Single-crystal X-ray diffraction data were collected on Bruker Quest D8 and Venture D8 single crystal X-ray diffractometers (Incoatec IμS, Mo *K*α, λ = 0.71073 Å). A light coating of paratone oil was used to affix the crystal to the mount and prevent exposure to the atmosphere and mitigate free water loss. All data collections were performed in a cold N_2_ gas stream to prevent crystal decomposition. Diffraction images were collected in 0.5° frame width using ϕ and ω scans. The data setup, collection, and processing were performed in the *APEX3* software suite (Bruker, 2015 ▸). Structure solution was accomplished using intrinsic phasing (*SHELXT*) and structure refinement was performed using full-matrix least-squares techniques on *F*^2^ (Sheldrick, 2015*a* ▸; Sheldrick, 2015*b* ▸). All non-hydrogen atoms were refined anisotropically. Hydrogen atoms attached to oxygen atoms were identified from the difference electron density maps and their positions refined using DFIX and DANG distance and angular geometric restraints. Substitutional disorder of Rb and Na atoms in the structure of **IV** was refined by free variable refinements of the atoms’ site occupancy factors. Crystallographic data are reported in Table 1 ▸. Selected interatomic distances are given in Table 2 ▸.

### 2.3. Magnetic property characterization

DC magnetization studies were performed on a co-aligned array of three hexagonal single crystals of K_5_[Fe_3_O(SO_4_)_6_(H_2_O)_3_]·5H_2_O (∼3 mg) using the vibrating sample magnetometry (VSM) option on a Quantum Design Physical Property Measurement System equipped with a 9 T (90 kOe) magnet. Crystals were preserved in rubber cement to retain moisture and affixed to a quartz paddle such the hexagonal *c* axis was oriented either parallel to or normal to the vertical applied magnetic field. Temperature-dependent studies were performed from 2 to 300 K in either zero-field-cooled or field-cooled modes under a static applied field. A 2 K min^−1^ ramp rate with 1 s averaging time of data points was used (one data point per 0.033 K). To model field dependence, isothermal field-dependent studies were performed from ±90 kOe with a 90 Oe s^−1^ sweep rate. The field was swept through the origin to assess potential hysteresis. Diamagnetic contributions from the holder and rubber cement were subtracted. Corrections due to Pascal’s constants were also performed (Bain & Berry, 2008 ▸).

## 3. Results and discussion

**Figure 3:**
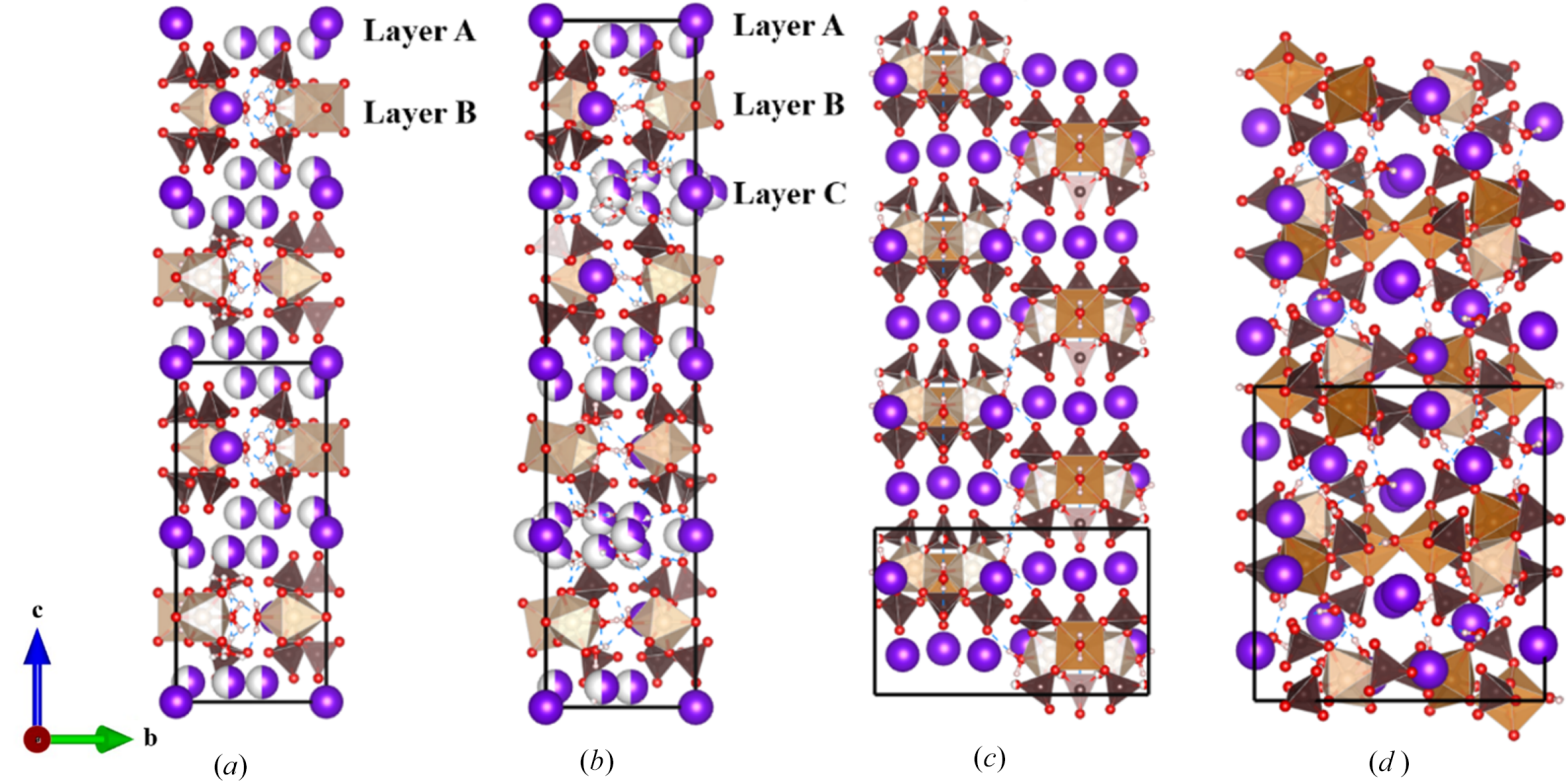
Layering comparison of unit cells for the Maus’s salt derivative compounds in the present study: (*a*) compound **I**, ∼19 Å *c* axis; (*b*) compound **II**, ∼39 Å *c* axis; (*c*) compound **III**, ∼9.7 Å *c* axis; (*d*) compound **IV**, ∼16 Å *c* axis. Color scheme is the same as in Fig. 1 ▸, with the addition of alkali metal ions shown as purple spheres (or partially purple partially white spheres for disordered sites).

Single crystals of the different Fe^3+^ compounds were prepared by a facile evaporative solution growth method, using various combinations of soluble alkali metal and iron sulfates, with H_2_O or mixed H_2_O/methanol as the solvent. Solutions were generally acidic despite the addition of weak sodium hydroxide due to the presence of Fe^3+^ ions. Crystal growth larger than 1 mm in diameter occurred for each product **I**–**V**, along with alkali-alum crystals that also grew in several cases. A series of Maus’s salt derivatives were obtained through exploratory synthesis using various alkali metal ions, representing several different space groups and structure types. Although large crystals are easily prepared for most of these derivatives, rapid degradation under ambient conditions proved problematic. As such, single-crystal X-ray diffraction data collections required low temperatures to stabilize the crystals. Based on our studies as well as previous studies, even minor variations in reaction conditions can lead to variations in the Maus’s salt structural motifs. The substitution of other alkali ions to expand the Maus’s salt phase space was conducted with Rb (**II**, **III**), Cs (**V**), and a mixed Na–Rb (**IV**) system. The latter mixture of ions is ideally a similar average ionic radius to potassium in the parent archetype, **I**. All the compounds except the Cs-containing product contain the same ferric trimer building blocks, with the arrangement of the alkali ions and hydrogen bonding of the water molecules leading to a very complex arrangement of stacking (Fig. 3 ▸, Table 3 ▸). While new structural derivatives with larger alkali ions were obtained, there was a clear pattern that substitution with larger alkalis resulted in a reduction of crystal symmetry as the alkali ion layering between iron trimers became more irregular and strained. Detailed discussion of compounds **I** to **V** will be given in their respective sections below.

### 3.1. Analysis of structures

#### 3.1.1. Crystal structure of K_5_[Fe_3_O(SO_4_)_6_(H_2_O)_3_]·5H_2_O, **I**

The prepared crystals, identified as the parent Maus’s salt structure, K_5_[Fe_3_O(SO_4_)_6_(H_2_O)_3_]·5H_2_O, crystallizes in the hexagonal space group *P*6_3_/*m*, aligning with the reported space group of K_2_(K_0.5_,H_2_O_0.5_)_6_(H_3_O)_2_Fe_3_(OH)_2_O(SO_4_)_6_·3H_2_O (Giacovazzo *et al.*, 1975 ▸). However, while this previous structural refinement identified the main features of the structure (Giacovazzo *et al.*, 1975 ▸), it lacked a full atom assignment. Herein, we pursued a low-temperature data collection to elucidate this largely ambiguous compound. These previous reports list a hexagonal unit cell with *a* = 9.71 (1) Å and *c* = 18.96 (2) Å, in relatively good agreement with our refinement (Table 1 ▸). The parent Maus’s salt structure reported in the current study is composed of one unique iron atom (6*h*) with two unique, half-occupied and disordered sulfur sites (12*i*) (we note that the sulfate group disorder could not be refined in an ordered arrangement by reducing the space group symmetry, or by introducing a *c*-axis supercell in test refinements). The formula of the parent compound, referred to as K-Maus’s salt, was originally reported as having a formula of K_2_(K_0.5_,H_2_O_0.5_)_6_(H_3_O)_2_Fe_3_(OH)_2_O(SO_4_)_6_·3H_2_O (Giacovazzo *et al.*, 1975 ▸). The original refinement assumes the requirement of two (H_3_O)^+^ groups due to position of water molecules on threefold sites. Therefore two (OH)^−^ groups were also reported for formula balancing. The formula reported here for **I** retains the same amount of K^+^ ions and bound H_2_O molecules but assigns only uncoordinated H_2_O molecules rather than any hydroxide or hydro­nium ions.

**Figure 4:**
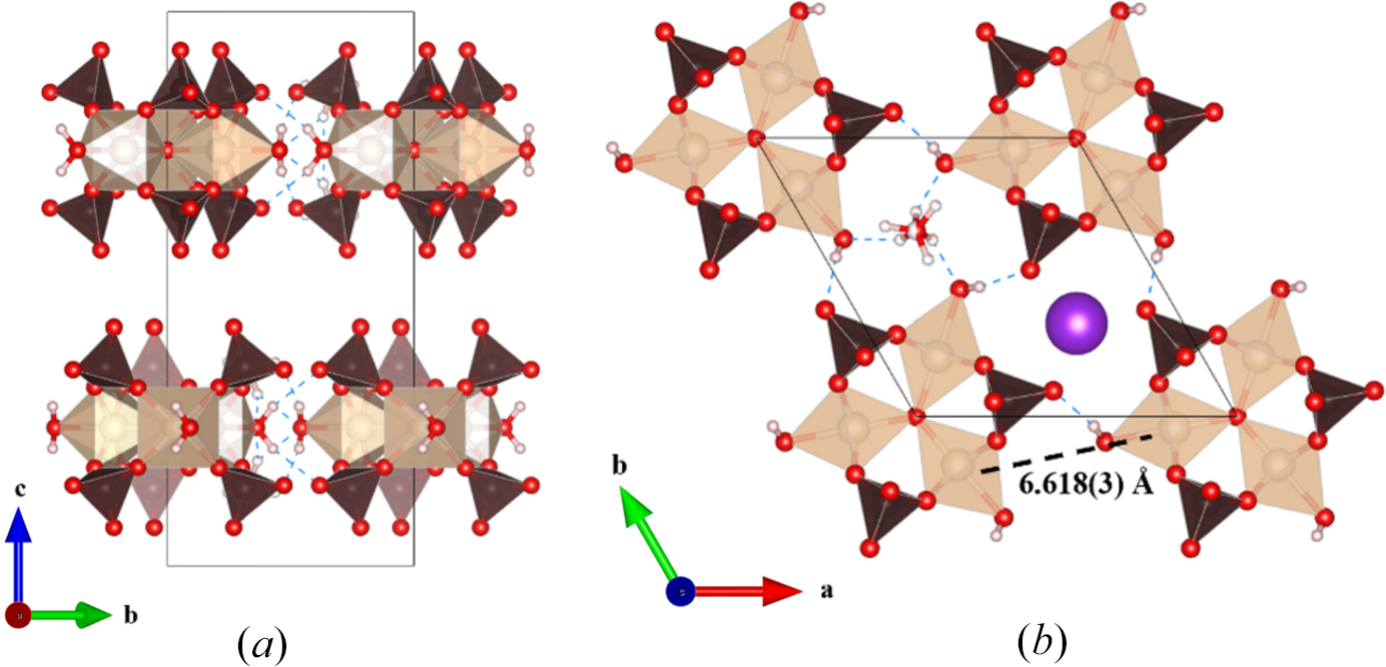
(*a*) Expanded unit cell of Fe trimers viewed down the *a* axis, (*b*) single layer hexagonal view of iron trimers forming channels containing water and the K3 atom, viewed along the *c* axis.

The most important structural feature of this compound is the planar ferric trimer (Fig. 1 ▸) centered by an oxygen atom with each six-coordinate iron center bridged by two bidentate sulfates and containing one water ligand which is in the waist site of the ring *trans* to the central oxide. The hydrogen atoms of these coordinated water ligands also contribute the hydrogen bonding of the water molecules bound to the K^+^ ions in the alkali metal layers of the structure (see below). The central oxygen atom, O5, has a distance to ferric ions of 1.9193 (12) Å, which is shorter than the Fe^3+^—O distance of 2.101 (9) Å for the Fe1—O6 to the coordinated water. These distances are within a typical distribution for Fe^3+^ (Bogatko *et al.*, 2010 ▸). The distance between these clusters is 6.6181 (3) Å along the *c* axis. Each ferric trimer is rotated by 60° in alternating layers (Fig. 4 ▸).

**Figure 5:**
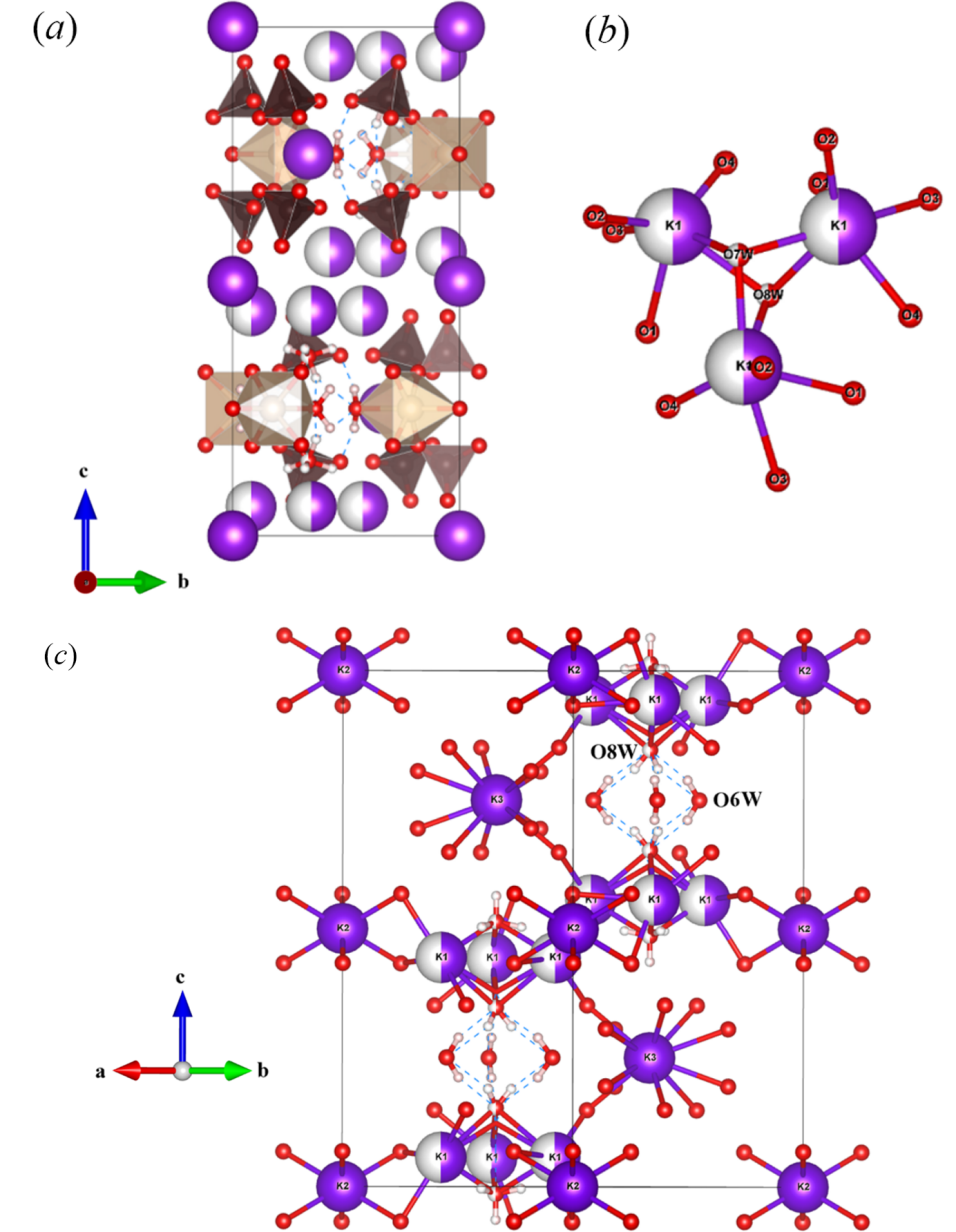
(*a*) Unit-cell contents of **I**; (*b*) coordination of partially occupied K1; (*c*) alkali coordination with water molecules and hydrogen bonding down the *c* axis, with K2 parallel to water channels, and K3 disordered along the water channel.

There are three unique K^+^ sites, namely the half-occupied K1, which was not definitively identified in the original structure, as well as fully occupied K2 and K3 sites which match the K2 and K3 sites in the original report (Giacovazzo *et al.*, 1975 ▸). There are two distinct layers that propagate normal to the *c* axis [Fig. 5 ▸(*a*)]. Layer *A* contains two different K sites, K1 and K2, while layer *B* is composed of K3 and the [Fe_3_O(SO_4_)_6_(H_2_O)_3_] groups. The K^+^ ions and their attendant water molecules have a very complex structural relationship, and this is essential to the full understanding of the structure of these Maus’s salts, so they are dealt with in some detail here. The most complex behavior involves the K1 ion. This site is only half occupied and located on a general position. It is coordinated by two half-occupied water ions, O7*W* within the *A* layer and O8*W* near the *B* layer, which reside on threefold symmetry axes along the *c* axis. This imposes trigonal disorder on the coordinated K1 ions (Fig. 5 ▸). This moiety in turn, is linked to itself by three water molecules [O6*W*] by hydrogen bonding. These O6 water molecules are those that are coordinated to the ferric ions of the iron cluster. This hydrogen bonding to the water ions coordinated to the disordered K1 ion plays a critical role in the structure of the parent Maus’s salt. We also believe that this disordered and partially occupied behavior of this potassium–water complex is likely a significant source of the instability of this compound. We suspect that variations in this region of the structure when different alkali metals and water contents are introduced play a major role in the wide variety of structural variations, some of which are discussed below.

The K2 ion resides in layer *A* as well but is well ordered and sits on a trigonal site. It features conventional six-coordination by O2 atoms, which also bridge to the K1 ions completing the two-dimensional structure in layer *A*. The remaining K^+^ ion, K3, also resides on a threefold site but occupies a position in layer *B*, the same layer that the ferric trimers occupy. It is also sixfold coordinated by water molecules that hydrogen bond in layer *B* to the bound O6*W* water molecule on Fe1. K3 is on a special position in the channel down the *c* axis, in agreement with previous reports, which also listed K—O interatomic distances between 2.7 and 3.2 Å (Mereiter & Völlenkle, 1978 ▸; Scordari, 1980 ▸; Giacovazzo *et al.*, 1975 ▸). The K1 and K2 atoms display similar K–O distances within this range.

#### 3.1.2. Crystal structure of Rb_5_[Fe_3_O(SO_4_)_6_(H_2_O)_3_]·5H_2_O, **II**

Another new Maus’s salt derivative, Rb_5_[Fe_3_O(SO_4_)_6_(H_2_O)_3_]·5H_2_O, crystallizes in trigonal space group *P*31*c*. While the *a* and *b* axes are similar to those in **I**, there is a notable difference with the *c* axis, effectively doubled to 38.798 (3) Å [Figs. 3 ▸(*b*) and 6 ▸]. The same [Fe_3_O(H_2_O)_3_(SO_4_)_6_] groups are observed in this compound, with the planar central oxygen, O5, having an Fe–O distance of 1.9169 (11) Å. The bound O10*W* facilitates the hydrogen bonding to oxygen site O6 on a neighboring cluster’s sulfate group (S2). This behavior propagates across the *ab* plane in the space between the clusters, with Rb2 also alternating in the space packing within the layer, which is designated as layer *B*.

In the structure of **II** there are a total of three unique layers normal to the *c* axis, hence the expanded *c* axis compared to **I**. Layers *A* and *C* are composed of multiple Rb^+^ ions along with coordinating water molecules sandwiching layer *B*. Layer *A* contains Rb1 and Rb4, while layer *C* is made up of Rb3, Rb5, and Rb6. Some of the alkali ions are fully occupied (Rb1, Rb2, Rb3) while several others are partially occupied, with half occupancy for Rb4 and one-third occupancy for Rb5 and Rb6. The fully occupied alkali sites adopt threefold symmetry sites, with Rb1 and Rb3 alternating down the *c* axis. The Rb4 ion behaves similar to the previously K1 atom in **I**, where the disorder is symmetry imposed. In **II**, the coordinated water molecules on the threefold axis, O11*W* and O12*W*, are fully occupied. The Rb5 and Rb6 ions also exhibit symmetry-imposed disorder. The arrangement of the alkali ions in layers *A* and *C* are slightly different from one another which results in the approximate doubling of the unit-cell axis along *c*. Rubidium coordination environments in **II** are shown in the supporting information, Fig. S1.

#### 3.1.3. Crystal structure of Rb_5_[Fe_3_O(SO_4_)_6_(H_2_O)_3_]·2H_2_O, **III**

**Figure 7:**
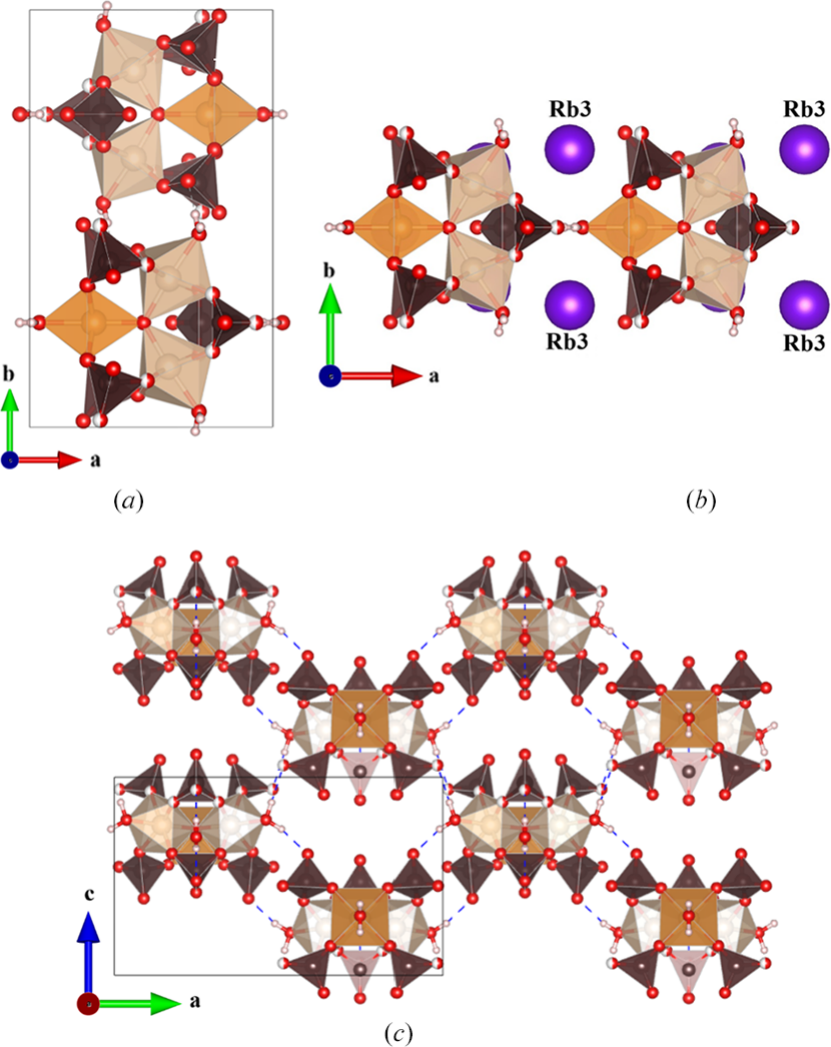
(*a*) View along *c* axis of alternating iron trimers in **III**, (*b*) Rb3 atoms between clusters, (*c*) hydrogen bonding through coordinated water molecules.

A second rubidium salt, Rb_5_[Fe_3_O(SO_4_)_6_(H_2_O)_3_]·2H_2_O, was isolated and refined in monoclinic space group *P*2_1_/*m*. Notably, this compound only contains two uncoordinated water molecules (that is, not coordinated to the Fe timers), compared to five in **I** and **II**. The loss of rigidly trigonal symmetry generates two unique iron sites in the trimer, with Fe1 and Fe2 in 4*f* and 2*e* Wyckoff sites, respectively, but the building blocks still possess the same essential structure. The coordinated water molecules hydrogen bond with the oxygen atoms of sulfates in neighboring clusters, which forms an intricate 2D pattern in the *ac* plane (Fig. 7 ▸).

**Figure 8:**
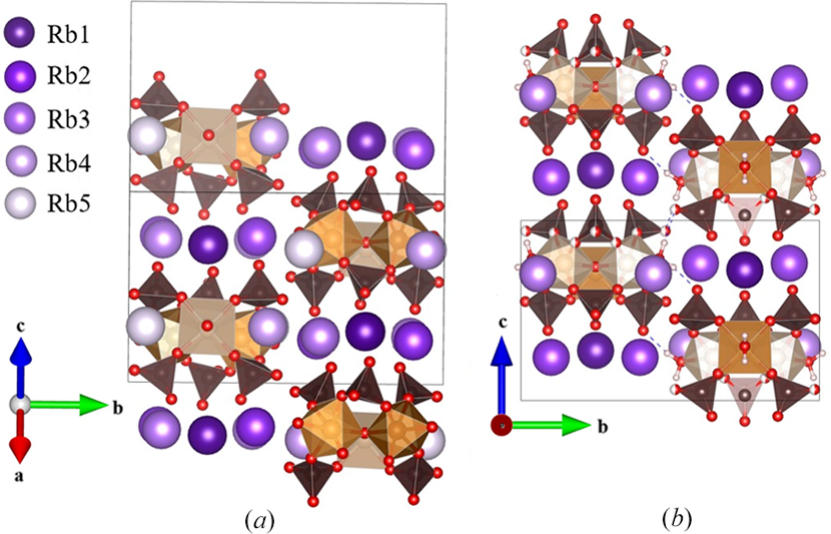
Alkali layering between clusters of (*a*) the supercell structure from the work of Mereiter & Völlenkle (1980 ▸) and (*b*) the unit cell of **III**.

There are several significant differences in the alkali ion and water layering here compared to most Maus’s salt derivatives. Likely because there are fewer water molecules in the lattice, the alkali ions in this structure are all well ordered with full site occupancy. Unlike most other Maus-type derivates, there are no continuous layers built only of hydrated alkali ions. The layering observed here has been previously reported for another Rb_5_[Fe_3_O(SO_4_)_6_(H_2_O)_3_]·2H_2_O polymorph with the following unit-cell parameters: *a* = 14.361 (3) Å, *b* = 16.033 (3) Å, *c* = 12.651 (3) Å, β = 92.04 (1)° (Mereiter & Völlenkle, 1980 ▸). This cell was the reported supercell of the compound, with a subcell identified as *a* = 9.40 Å, *b* = 16.03 Å, *c* = 9.74 Å, β = 97.3°. Their subcell effectively corresponds to compound **III** of the current study. We did not obtain a satisfactory refinement by considering a supercell for **III** to match this previous report. Mereiter & Völlenkle (1980 ▸) found that the subcell could be observed with a variety of mixed alkali cations, rather than exclusively five Rb^+^ ions [Fig. 8 ▸(*a*)]. Our unit cell indeed contains five Rb^+^ ions per formula unit with good refinement in three unique positions [Fig. 8 ▸(*b*)] (the supercell accounts for the five alkali metal ions in the formula unit through five unique sites). In **III**, the Rb1 and Rb2 sites occupy the parallel space down the *c* axis, while Rb3 fills space between neighboring clusters along the *a* axis (Fig. 8 ▸) and is coordinated by water O16*W*. The overall result is a staggered arrangement of columns of alternating layers in **III**, rather than alternating continuous layers in **I** and **II**. The water molecules coordinated to Fe^3+^ give rise to different structural behavior than in **I** and **II**, where they all formed hydrogen bonds between clusters. In **III**, Rb3 is interacting with both O17*W* and O18*W*. The O17*W* atom hydrogen bonds to O9 on the sulfate of a neighboring cluster. The two remaining water molecules in the formula coordinate to the alkali ions, with many of these oxygen atoms bridged across multiple unique Rb sites (Fig. S2).

#### 3.1.4. Synthesis and crystal structure of (Na_1.8_Rb_3.2_)[Fe_3_O(H_2_O)_3_(SO_4_)_6_]·5H_2_O, **IV**

We attempted to prepare other examples of Maus’s salt with trigonal symmetry analogous to the parent potassium salt to study additional structures comprised of these trigonal magnetic trimer units. In this regard we examined several Na^+^/Rb^+^ ratios hoping to mimic an average ionic ratio similar to that of K^+^ and, in the process, obtained **IV** instead. There have been several reports of Maus’s salt structure types containing multiple mixed alkali ions but with structures very different from that reported here (Scordari, 1980 ▸; Mereiter & Völlenkle, 1980 ▸; Mereiter, 2013 ▸). Our site refinement of **IV** suggests an approximate 1.8Na/3.2Rb ratio (supporting information, Table S1), which is similar to previously reported mixed alkali ion salts. It forms in a relatively low-symmetry *P*2_1_/*n* space group and has axis lengths [*a* = 12.8404 (6) Å, *b* = 14.7424 (6) Å, *c* = 16.1710 (7) Å] which, interestingly, are similar to the supercell lengths previously reported (Mereiter & Völlenkle, 1980 ▸), but the beta angle in our case is quite different [β = 99.948 (2)° versus 92.01 (1)°] as is the overall structure.

**Figure 9:**
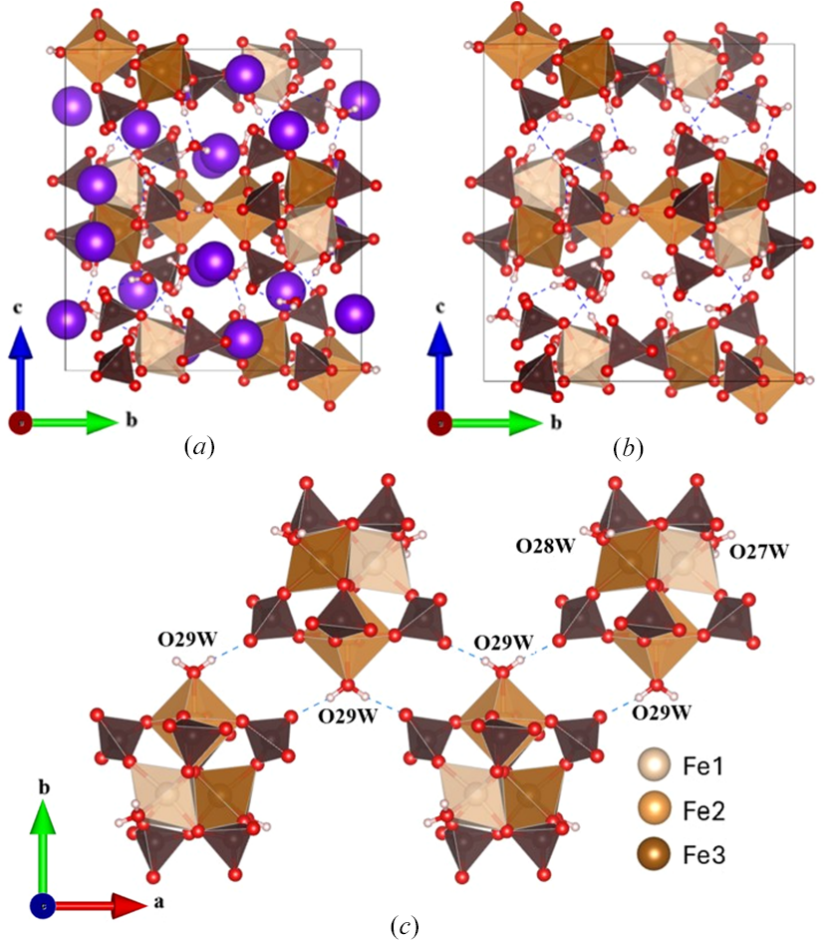
(*a*) Iron trimer clusters with mixed Na/Rb sites occurring between clusters in **IV**, (*b*) iron trimer clusters with water molecules in **IV** (Na/Rb sites omitted for clarity), (*c*) staggered connectivity of iron trimer clusters in **IV** occurring through the coordinated water molecules’ interactions with neighboring sulfate groups.

In **IV** the basic building block of sulfate-bridging ferric trimers is still present (Fig. 9 ▸), but their packing behavior is quite different from the various other K^+^ and Rb^+^ ion types in **I**–**III**. In this case there are four ferric trimers in the unit cell, and they form a pseudo-threefold packing arrangement across the *ab* diagonal. The clusters hydrogen bond to each other via their coordinated water molecules to form larger scale layers but these layers are different from the classic Maus’s salt layers in that they are not planar. They do form a stacking arrangement with columns of trimers down the *ac* diagonal. An important structural feature between these clusters is found regarding the alkali ions, different from our other Rb derivatives, **II** and **III**. Rather than being composed of an alkali channel, as in **II**, or ordered layering of alkali ions between layers of iron trimers, as in **III**, the alkali ion occupy space between the trimers with the parallel space occupied by water molecules (Fig. 9 ▸). Each of these alkali sites have mixed occupancy, with varying percentages of Na and Rb. The offset cluster packing pattern is similar to that of **III**, with the difference being the location of the alkali ions and the increased water content in the columns. The trimers themselves form hydrogen bonding interactions between one another via the coordinated water molecules.

**Figure 10:**
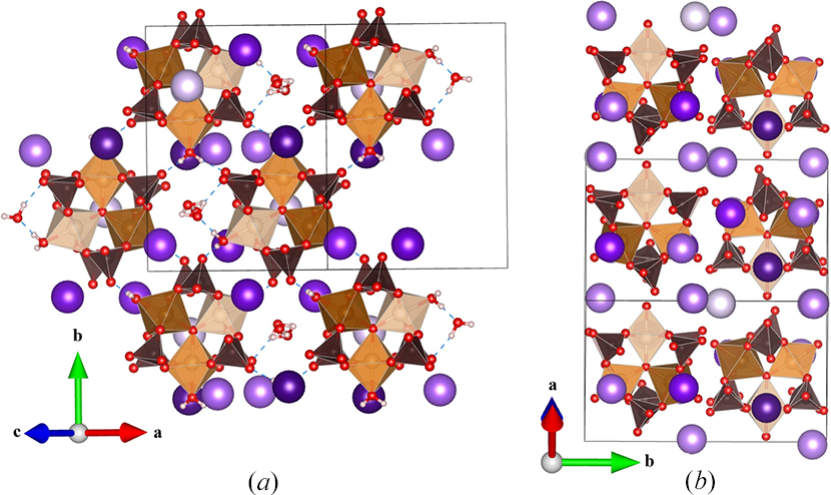
(*a*) Crystal structure of **IV** viewed along the pseudo-threefold symmetry axis of the iron trimers. (*b*) Crystal structure derived from the work of Mereiter & Völlenkle (1980 ▸) also viewed along the pseudo-threefold symmetry axis of the iron trimers.

As discussed above, the alkali and uncoordinated water molecules can drive the formation of a wide range of structures, and this is no exception. It can be compared to the previously reported *P*2_1_/*n* structure of Rb_5_[Fe_3_O(SO_4_)_6_(H_2_O)_3_]·2H_2_O, that exhibits similar unit-cell axis values but with a different beta angle (Mereiter & Völlenkle, 1980 ▸). Despite having similar unit-cell axis parameters and some sodium ions in place of several larger rubidium ions, this compound has a significantly larger cell volume [3015.1 (2) Å^3^ versus 2911.05 Å^3^] to accommodate the additional non-coordinated water content of **IV**. The pseudo-trigonal view of these two compounds gives some insight into the unit cell packing, with **IV** having distinct water channels down the *ac* diagonal (Fig. 10 ▸). These channels are shared with Rb2 and Rb3 between neighboring clusters. The remaining alkali sites, Rb1, Rb4 and Rb5, lie between the layers of iron trimers. Compound **III**, and the related supercell (Mereiter & Völlenkle, 1980 ▸), both lack a distinct water channel, with the main trimer separation being attributed to alkali bonding distances.

#### 3.1.5. Crystal structure of Cs_5_[Fe_4_O_2_(HSO_4_)(SO_4_)_6_(H_2_O)_3_]·1.75H_2_O, **V**

**Figure 11:**
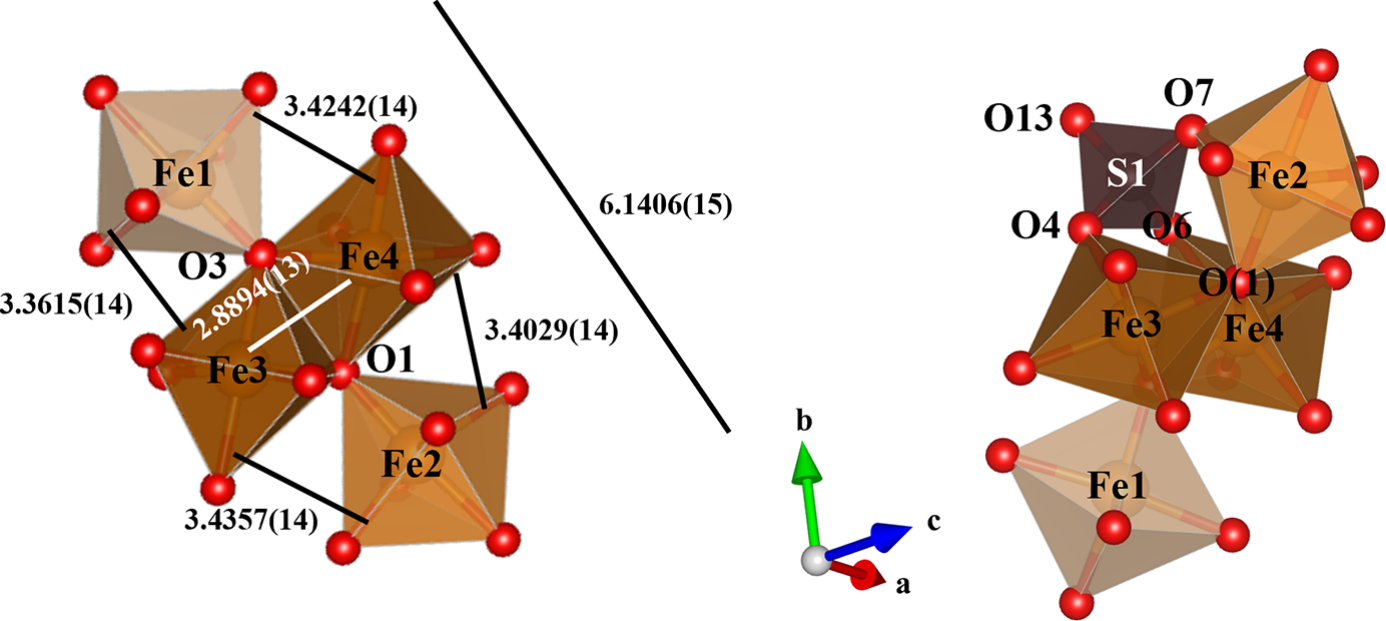
Iron tetramers in the structure of **V**: (*a*) connectivity via edge and corner sharing of octahedra, (*b*) tetramer capping by S3 coordination to three iron sites.

**Figure 12:**
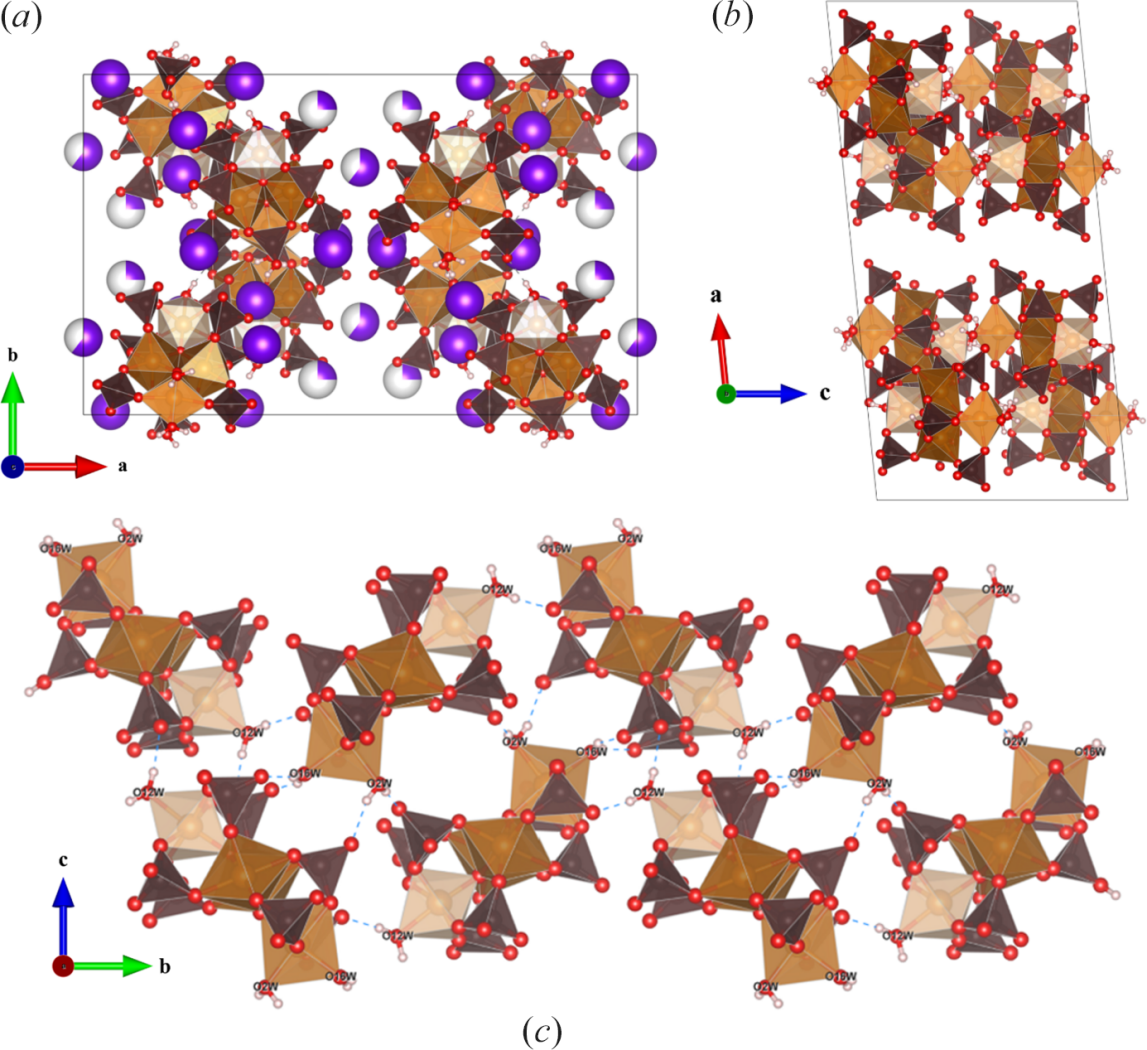
Structure of **V**: (*a*) unit-cell contents viewed along the *c* axis, (*b*) formation of tetramer layers viewed along the *b* axis, (*c*) hydrogen bonding between tetramer clusters viewed along the *a* axis.

In the continued investigation of the role of alkali ions we attempted to form Cs^+^ analogs of Maus’s salts. To our knowledge there has only been one reported Maus-type compound containing Cs^+^ ions, namely the unusual Cs_2.91_Na_1.34_Fe^3+^_0.25_[Fe_3_O(SO_4_)_6_(H_2_O)_3_]·5H_2_O (Mereiter, 2013 ▸) which displays mixed occupancy of Cs, Na and Fe^3+^. That structure follows the general Maus’s salt type with characteristic trimers. To test the effect of Cs^+^ ions we did the usual synthetic reactions with Cs^+^ as the only alkali present. We were surprised to isolate a very different compound with the formula Cs_5_[Fe_4_O_2_(HSO_4_)(SO_4_)_6_(H_2_O)_3_]·1.75H_2_O. This structure adopts space group *C*2/*c*, with eight unique clusters in the large unit cell. The tetrameric cluster is built of octahedrally coordinated Fe^3+^ with edge-sharing dimers [Fe3 and Fe4 with a distance of 2.8894 (14) Å], where edge-bridging oxides are also corner bridged to Fe1 and Fe2 octahedra. The clusters are decorated by capping of seven sulfate tetrahedra and three coordinated water molecules. This ferric tetramer structure type is well known among iron sulfate minerals such as hohmanite (Fe_2_(H_2_O)_4_[(SO_4_)_2_O]·4H_2_O) (Scordari, 1978 ▸) and amarantite (Fe_2_(H_2_O)_4_[(SO_4_)_2_O]·3H_2_O) (Süsse, 1968 ▸). The primary difference is that the mineral species have extended polymeric chains linked by the sulfates with hydrogen bonding linking parallel chains, while **V** is built of isolated tetrameric clusters again interacting through a complex network of hydrogen bonds from various water molecules as in the Maus’s salts. The corner-sharing iron atoms have a range of 3.3615 to 3.4357 (14) Å to the dimer (Fe3 and Fe4), and 6.1406 (15) Å to one another (Fig. 11 ▸). The S3 atom has three basal oxygens (O4, O6, O7) shared with three of the iron atoms (Fe2, Fe3, and Fe4) of a given tetramer, while the rest of the sulfur sites only share two oxygen atoms with any combination of two iron sites. The apex oxygen atom of the S3 tetrahedron supports H13 to form the [HSO_4_]^−^ group. A total of three water molecules are coordinated to Fe1 and Fe2 atoms in the tetramers. The Fe1 site coordinates O32*W* and the Fe2 site coordinates O31*W* and O33*W* in a *cis* arrangement. The Fe3 and Fe4 atoms that are edge sharing in the tetramer are not coordinated by water molecules. As in the Maus’s salts, there is hydrogen bonding between neighboring clusters originating from the iron-coordinated water molecules to the sulfate oxygen atoms of adjacent clusters (Fig. 12 ▸, Table S2). This propagates the clusters in a spiral arrangement down the *b* axis. Four fully occupied Cs sites are coordinated by oxygen atoms of the sulfate tetrahedra with additional disordered caesium ions and water molecules located in channels/voids oriented along the *c* axis, between tetramer clusters.

It is not obvious why the caesium ferric sulfate forms such a different building block from that of the Maus’s salts analogs, other than it is larger than the other alkali ions. It is not surprising, however, given the obvious importance of even subtle changes in alkali ion size and water coordination in creating the packing in Maus’s salts. Between the considerable lability of the ferric clusters and the driving force of the hydrogen bonding in forming the structures, the stabilization of a different structure type is not unexpected.

### 3.2. DC magnetization studies of K_5_[Fe_3_O(SO_4_)_6_(H_2_O)_3_]·5H_2_O, **I**

**Figure 13:**
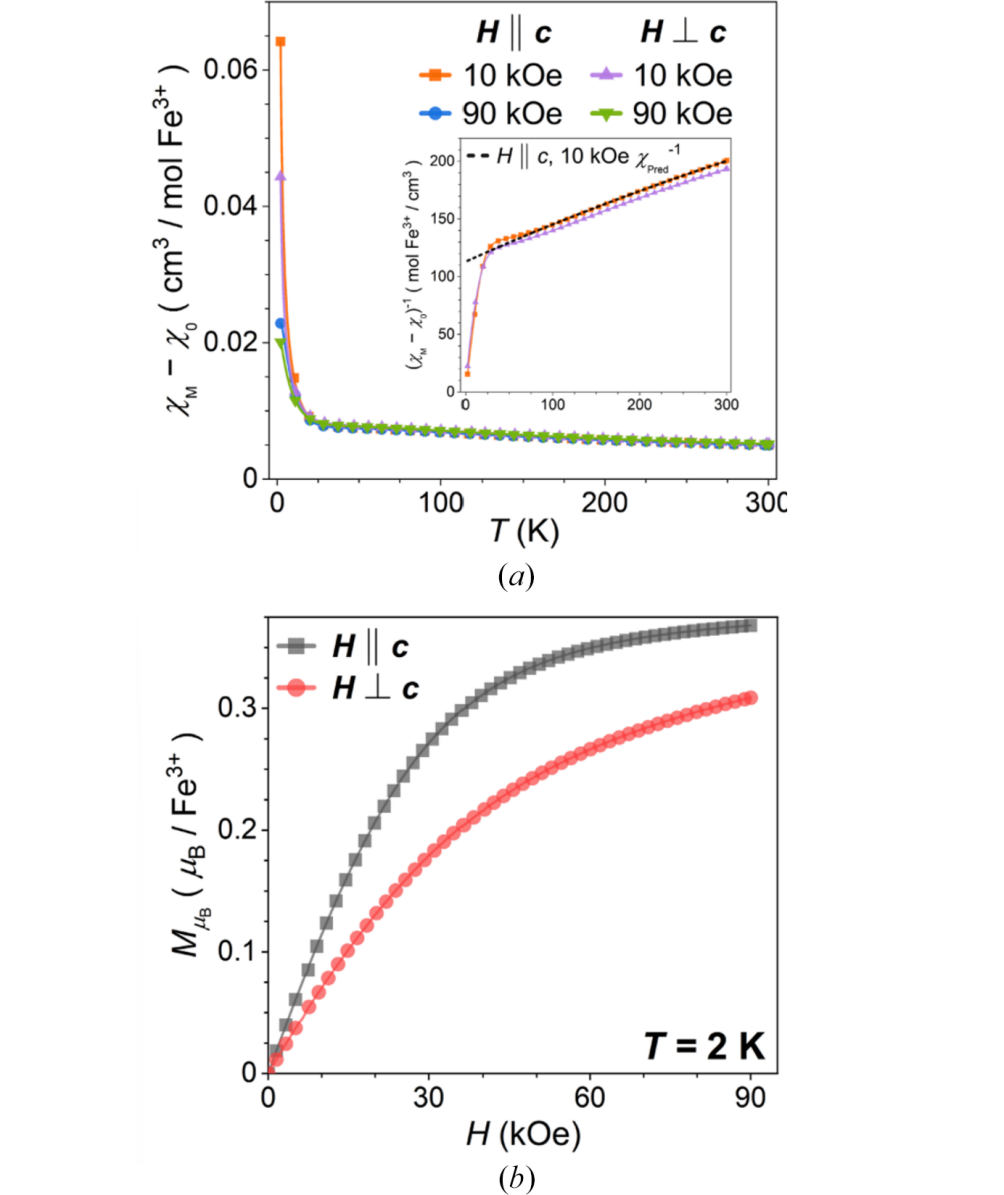
(*a*) Orientation- and field-dependent magnetic susceptibility of K_5_[Fe_3_O(SO_4_)_6_(H_2_O)_3_]·5H_2_O (**I**) relative to the trigonal *c* axis (inset: inverse susceptibility curves and illustrative Curie–Weiss fit (dashed-line) demonstrating an antiferromagnetic deflection in the 20–100 K range); (*b*) magnetization curves at 2 K.

The trigonal arrangement of metal sulfate trimers in the Maus’s salt family offers the potential for magnetic frustration if the predominant interactions are AFM and degenerate ground state orientations are assumed. In the case of the parent material K_5_[Fe_3_O(SO_4_)_6_(H_2_O)_3_]·5H_2_O, there is trigonal symmetry both within and between the planar trimers leaving several spin-exchange coupling pathways for magnetic frustration to arise. To gain initial insights into this possibility, magnetization studies were performed. Since the crystal can be grown in fairly large sizes with obvious uniaxial geometry, their anisotropic behavior can easily be probed through axis-oriented magnetization studies in various applied magnetic fields. The magnetic susceptibility of **I** displays an extremely subtle broad hump with local maximum near 60–70 K attributed to short-range reorientation of Fe^3+^ spins within the trimers [Fig. 13 ▸(*a*)]. This feature was found in both the perpendicular (⊥) and parallel (∥) orientations relative to the *c* axis. There is a minor effect related to field strength, with the 90 kOe curves having a slightly more pronounced curvature around the local maxima near 60–70 K. No effect was seen between cooling methods in an applied field or lack thereof, so only the zero field cooled data are shown. Isothermal field-dependent magnetization curves have the appearance of magnetic saturation with a moment an order-of-magnitude lower than the expected 5 μ_B_. Slight easy-hard anisotropy and *g*-tensor anisotropy is seen in the respective low- and high-field regimes by the separation of isotherms. Through these preliminary magnetization studies, the proposed magnetic structure is likely a splayed structure with spin moments predominantly in the trimer plane and oriented about the local *z* axis (bound water and central oxygen axis) of each octahedra. The low saturation moment implies the spin moments point inwards towards the central shared oxygen. The spins are more susceptible to spin torquing normal to this plane, suggestive of slightly stronger intra-planer interactions versus those out of the plane.

To gain insights into the general high-temperature magnetic behavior of the high-spin, *S* = 5/2 Fe^3+^ trimers, the curves were fitted with the Curie–Weiss (CW) model in the 200–300 K linear range of the inverse susceptibility [inset, Fig. 13 ▸(*a*)]. While the CW model does not strictly apply in cases of non-isotropic systems let alone one with anisotropic heteroligand octahedra (Van Vleck, 1973 ▸), the fit is provided solely as a first approximation of pertinent parameters. Fig. S4 illustrates the resultant fit and Table 4 ▸ reports the pertinent parameters. These orientation-dependent measurements also give insight into the respective *g*-tensor components relative to the threefold crystallographic *c* axis. For both the CW temperature, Θ_CW_, and Curie constant, *C*, the fitted values perpendicular to the *c* axis were nearly 30% lower than the respective values when the applied field is oriented parallel to the trigonal axis, further suggestion of *g*-tensor anisotropy relative to the Fe^3+^ trimers. These data suggest the spin moments have a greater tendency to orient themselves towards the *c* axis but still have a significant canted component that helps explain the short-range AFM reorientation event occurring near 60–70 K. The estimated moments along either direction are also below the expected value of 5.92 μ_B_ for high-spin Fe^3+^. The Θ_CW_ values are all highly negative with a 100 K difference between orientations. By this parameter, the interactions in the plane of the trimers (∥) would then be expected to be more AFM than the ones normal to the plane (⊥) by approximately 40%. Again, the structural anisotropy and the lack of 1/*T*-like behavior in the susceptibility curves again requires a qualitative view of the obtained CW parameters. The local through-space and superexchange coupling within trimer units could be the source of this anisotropy and the subtle rollover feature. Goodenough–Kanamori–Anderson rules imply weak FM superexchange through the central oxygen of the trimers with very weak FM interactions through ∼90° super-superexchange mediated by the sulfate groups, which could give rise to hypothesized spin canting behavior from competing orientations in FM interactions. Subtle deviations in the alkali positions and distortions in the sulfates could further perturb these weak coupling interactions.

Heat capacity measurements on a 0.6 g single crystal with 0 Oe and 90 kOe fields parallel to the *c* axis revealed no notable features, matching the susceptibility curves with the lack of a clear AFM ordering transition and strong spin reorientations (Fig. S5). The most significant feature was a subtle difference in low-temperature profiles, possibly hinting at field-induced spin torquing as thermal energy is frozen out. Neutron scattering studies would be instrumental in discovering the nature of the spin moments within the trigonal arrangement of Fe^3+^ centers.

## 4. Conclusions

Several interesting results emerged from this study. The first is that we were able to obtain high-quality single-crystal structures of several Maus’s salt derivatives, including the parent material, for the first time using data obtained at low temperatures while taking care to prevent crystal dehydration. It is clear that the solvation sphere of the alkali ions and the hydrogen bonding between the various components play an essential role in the structural formation of these species. These low-temperature data collections provide what we hope is the definitive structure of the parent Maus’s salt first reported nearly 200 years ago. The low-temperature data maintains the integrity of the unit cell, helps determine the complex structural features (especially the hydrogen-bonding), and resolves some of the ambiguities of the various structural reports in the past. In efforts to study the hydration sphere around the alkali and hydrogen bonding through variation of alkali identity, we obtained several new structural variations of rubidium-containing Maus’s salt and a new caesium-based derivative. Given the importance of the subtle aspects of the alkali aqua­tion spheres, it is not surprising that the caesium derivative leads to an entirely different structure type, built of ferric tetramers rather than the characteristic Maus’s salts trimers. It should be clear however that other yet-undiscovered synthetic routes may still lead to Cs^+^ Maus-type structures. We do not think that we have isolated all the structural variations within this complex class of materials. With the small chemical changes leading to complex structural changes, it is clear that many structural types still await discovery. Finally, one of our driving forces to develop this chemistry was the possibility of obtaining unusual magnetic behavior as observed in other ferric sulfates such as jarosite (Nocera *et al.*, 2004 ▸). In this case we were encouraged by the trigonal symmetry both within and between the iron trimer building blocks of the parent Maus’s salt. Clear indications of magneto-structural anisotropy and possible magnetic frustration are present through the low saturation moment in field-dependent magnetization measurements, which imply a splayed magnetic structure within the trimer units with spin moments pointing inward about the local *z* axis towards the central shared oxygen. As such, more detailed studies are needed to understand the magnetic behavior and the relative strength of intra-trimer superexchange or inter-trimer dipole–dipole spin-coupling interactions play in this canted state.

## Supplementary Material

Crystal structure: contains datablock(s) I, II, III, IV, V. DOI: 10.1107/S2052520626002027/tq5033sup1.cif

Structure factors: contains datablock(s) I. DOI: 10.1107/S2052520626002027/tq5033Isup2.hkl

Structure factors: contains datablock(s) II. DOI: 10.1107/S2052520626002027/tq5033IIsup3.hkl

Structure factors: contains datablock(s) III. DOI: 10.1107/S2052520626002027/tq5033IIIsup4.hkl

Structure factors: contains datablock(s) IV. DOI: 10.1107/S2052520626002027/tq5033IVsup5.hkl

Structure factors: contains datablock(s) V. DOI: 10.1107/S2052520626002027/tq5033Vsup6.hkl

Tables S1 and S2, Figs. S1-S5. DOI: 10.1107/S2052520626002027/tq5033sup7.pdf

CCDC references: 2532594, 2532595, 2532596, 2532597, 2532598

## Figures and Tables

**Figure 6:**
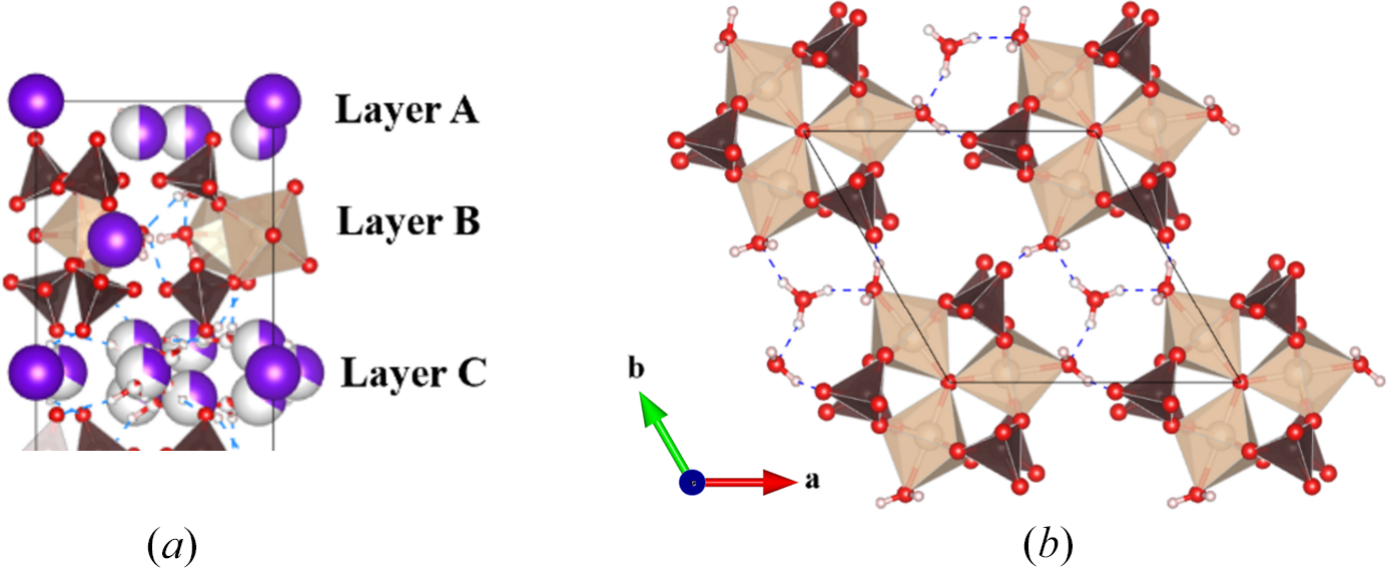
(*a*) Partial structural view of the three unique layers stacking along the *c* axis in **II**, (*b*) threefold symmetry of trimers viewed down the *c* axis, including hydrogen bonding of stacked water molecules in the channels (Rb atoms omitted for clarity).

**Table:** 

	**I**	**II**	**III**
Crystal data			
Chemical formula	K_5_[Fe_3_O(SO_4_)_6_(H_2_O)_3_]·5H_2_O	Rb_5_[Fe_3_O(SO_4_)_6_(H_2_O)_3_]·5H_2_O	Rb_5_[Fe_3_O(SO_4_)_6_(H_2_O)_3_]·2H_2_O
*M*_r_	1099.54	1331.39	1277.34
Crystal system, space group	Hexagonal, *P*6_3_/*m*	Trigonal, *P* 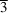 1*c*	Monoclinic, *P*2_1_/*m*
Temperature (K)	100	100	136
*a*, *b*, *c* (Å)	9.6605 (3), 9.6605 (3), 18.7613 (9)	9.7444 (4), 9.7444 (4), 38.798 (3)	9.3673 (2), 15.9584 (4), 9.7171 (3)
α, β, γ (°)	90, 90, 120	90, 90, 120	90, 97.1001 (9), 90
*V* (Å^3^)	1516.33 (12)	3190.5 (3)	1441.44 (7)
*Z*	2	4	2
Radiation type	Mo *K*α	Mo *K*α	Mo *K*α
μ (mm^−1^)	2.64	9.43	10.43
Crystal size (mm)	0.12 × 0.06 × 0.04	0.17 × 0.15 × 0.11	0.08 × 0.06 × 0.05

Data collection			
Diffractometer	Bruker D8 Quest Photon 3	Bruker D8 Quest Photon 3	Bruker D8 Quest Photon 3
Absorption correction	Multi-scan (*SADABS 2014/5*)	Multi-scan (*SADABS 2014/5*)	Multi-scan (*SADABS 2014/5*)
*T*_min_, *T*_max_	0.911, 1.000	0.865, 1.000	0.822, 1.000
No. of measured, independent and observed [*I* > 2σ(*I*)] reflections	28963, 965, 857	68059, 2095, 1812	35524, 2843, 2492
*R*_int_	0.077	0.090	0.067
(sin θ/λ)_max_ (Å^−1^)	0.603	0.617	0.610

Refinement			
*R*[*F*^2^ > 2σ(*F*^2^)], *wR*(*F*^2^), *S*	0.052, 0.142, 1.17	0.043, 0.120, 1.05	0.048, 0.106, 1.12
No. of reflections	965	2095	2843
No. of parameters	140	192	265
No. of restraints	32	24	73
H-atom treatment	H atoms treated by a mixture of independent and constrained refinement	H atoms treated by a mixture of independent and constrained refinement	H atoms treated by a mixture of independent and constrained refinement
Δρ_max_, Δρ_min_ (e Å^−3^)	0.90, −0.85	1.52, −1.32	1.49, −1.28

**Table:** 

	**IV**	**V**
Crystal data		
Chemical formula	(Na_1.8_Rb_3.2_)[Fe_3_O(H_2_O)_3_(SO_4_)_6_]·5H_2_O	Cs_5_[Fe_4_O_2_(HSO_4_)(SO_4_)_6_(H_2_O)_3_]·1.75H_2_O
*M*_r_	1219.39	1678.95
Crystal system, space group	Monoclinic, *P*2_1_/*n*	Monoclinic, *C*2/*c*
Temperature (K)	100	100
*a*, *b*, *c* (Å)	12.8404 (6), 14.7424 (6), 16.1710 (7)	31.105 (4), 19.033 (2), 15.5664 (19)
α, β, γ (°)	90, 99.948 (2), 90	90, 95.798 (4), 90
*V* (Å^3^)	3015.1 (2)	9168.5 (19)
*Z*	4	8
Radiation type	Mo *K*α	Mo *K*α
μ (mm^−1^)	7.13	5.56
Crystal size (mm)	0.11 × 0.08 × 0.04	0.12 × 0.10 × 0.08

Data collection		
Diffractometer	Bruker D8 Venture Photon 2	Bruker D8 Venture Photon 2
Absorption correction	Multi-scan (*SADABS 2014/5*)	Multi-scan (*SADABS 2014/5*)
*T*_min_, *T*_max_	0.842, 1.000	0.755, 1.000
No. of measured, independent and observed [*I* > 2σ(*I*)] reflections	63096, 6237, 5147	134104, 9499, 8164
*R*_int_	0.066	0.064
(sin θ/λ)_max_ (Å^−1^)	0.628	0.628

Refinement		
*R*[*F*^2^ > 2σ(*F*^2^)], *wR*(*F*^2^), *S*	0.029, 0.068, 1.07	0.042, 0.118, 1.06
No. of reflections	6237	9499
No. of parameters	477	566
No. of restraints	24	60
H-atom treatment	Only H-atom coordinates refined	H atoms treated by a mixture of independent and constrained refinement
Δρ_max_, Δρ_min_ (e Å^−3^)	1.11, −0.76	2.37, −1.44

Computer programs: *APEX3* (Bruker, 2017 ▸), *SADABS* (Krause *et al.*, 2015 ▸), *SAINT* (Bruker, 2015 ▸), *SHELXT 2014/5* (Sheldrick, 2015*a* ▸), *SHELXL2016/6* (Sheldrick, 2015*b* ▸), *Vesta 3* (Momma *et al.* 2011 ▸), *publCIF* (Westrip, 2010 ▸).

**Table 2:** Interatomic Fe—O distances (Å) for Maus’s salt derivatives **I**–**IV**

**I**		**II**		**III**		**IV**	
Fe1—O5	1.9189 (10)	Fe1—O5	1.9184 (9)	Fe1—O5	1.934 (3)	Fe1—O5	1.907 (2)
Fe1—O3 ×2	1.950 (8)	Fe1—O4	1.967 (5)	Fe1—O14	1.993 (9)	Fe1—O2	2.002 (2)
Fe1—O1 ×2	2.003 (11)	Fe1—O8	1.988 (5)	Fe1—O15	2.004 (9)	Fe1—O21	2.026 (2)
Fe1—O6*W*	2.097 (6)	Fe1—O7	2.013 (5)	Fe1—O11	1.971 (5)	Fe1—O10	1.999 (2)
		Fe1—O3	2.031 (5)	Fe1—O12	1.996 (6)	Fe1—O1	2.008 (2)
		Fe1—O10*W*	2.091 (6)	Fe1—O17*W*	2.070 (5)	Fe1—O27*W*	2.108 (2)
				Fe2—O5	1.914 (7)	Fe2—O5	1.944 (2)
				Fe2—O8 ×2	1.995 (5)	Fe2—O4	2.006 (2)
				Fe2—O1 ×2	2.013 (5)	Fe2—O19	2.007 (2)
				Fe2—O18*W*	2.077 (7)	Fe2—O17	2.002 (2)
						Fe2—O12	2.007 (2)
						Fe2—O29*W*	2.085 (2)
						Fe3—O5	1.923 (2)
						Fe3—O8	2.005 (2)
						Fe3—O7	2.020 (2)
						Fe3—O11	1.994 (2)
						Fe3—O6	2.020 (2)
						Fe3—O28*W*	2.045 (3)

**Table 3:** Hydrogen-bonding interactions (Å, °) originating from iron-coordinated water molecules in Maus’s salt related compounds **I**–**IV**

Compound	*D*—H⋯*A*	*d*(O—H)	*d*(H⋯O)	*d*(O⋯O)	∠(O–H⋯O)	Acceptor atom symmetry code
**I**	O6*W*—H⋯O4	0.92 (2)	1.87 (3)	2.761 (18)	164 (6)	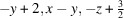
**I**	O6*W*—H⋯O4	0.92 (2)	1.87 (3)	2.761 (18)	164 (6)	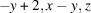
**II**	O10*W*—H⋯O6	0.90 (2)	2.08 (6)	2.709 (7)	126 (6)	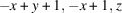
**II**	O10*W*—H⋯O7	0.90 (2)	2.21 (8)	2.850 (10)	128 (8)	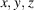
**III**	O17*W*—H⋯O9	0.89 (2)	1.75 (6)	2.648 (7)	172 (7)	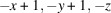
**III**	O17*W*—H⋯O13	0.89 (2)	1.91 (4)	2.801 (14)	175 (7)	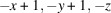
**III**	O18*W*—H⋯O6	0.90 (2)	1.77 (6)	2.653 (7)	167 (7)	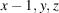
**III**	O18*W*—H⋯O4	0.90 (2)	1.86 (6)	2.714 (7)	158 (7)	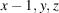
**IV**	O27*W*—H⋯O26*W*	0.895 (19)	1.828 (19)	2.717 (4)	172 (5)	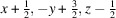
**IV**	O27*W*—H⋯O23	0.881 (19)	1.85 (2)	2.728 (3)	175 (5)	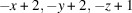
**IV**	O28*W*—H⋯O33*W*	0.889 (19)	1.76 (2)	2.644 (4)	177 (5)	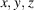
**IV**	O28*W*—H⋯O25	0.878 (19)	1.80 (2)	2.670 (3)	174 (5)	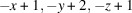
**IV**	O29*W*—H⋯O20	0.887 (19)	1.80 (2)	2.671 (3)	169 (5)	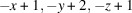
**IV**	O29*W*—H⋯O14	0.886 (19)	1.87 (2)	2.740 (3)	168 (5)	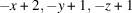

**Table 4:** Curie–Weiss fit parameters modeled in the 200–300 K range of the 10 kOe inverse susceptibility curves (see Fig. S4)

	Θ_CW_ (K)	*C* (cm^3^ K mol^−1^ Fe^3+^)	μ_eff_ (μ_B_)
10 kOe ∥ *c*	−279 (4)	2.08 (3)	4.08
90 kOe ∥ *c*	−255 (3)	1.88 (2)	3.88
10 kOe ⊥ *c*	−188 (2)	1.46 (2)	3.42
90 kOe ⊥ *c*	−156 (2)	1.23 (1)	3.14
