# Supplementary material for: Maus’s salts: an old family of iron sulfates with possible magnetic frustration
Source: Acta Crystallogr B Struct Sci Cryst Eng Mater. 2026 May 7;82(Pt 3):268–79. doi: 10.1107/S2052520626002027 (PMC13238487; doi:10.1107/S2052520626002027)
Supplement: Supplementary file 7 [file b-82-00268-sup7.pdf]

Acta Cryst B

STRUCTURAL SCIENCE
CRYSTAL ENGINEERING
MATERIALS

Volume 82 (2026)

Supporting information for article:

Maus's salts: an old family of iron sulfates with possible magnetic frustration

Analeece Long, Matthew Powell, Ashley Dickey, Colin McMillen and Joseph Kolis

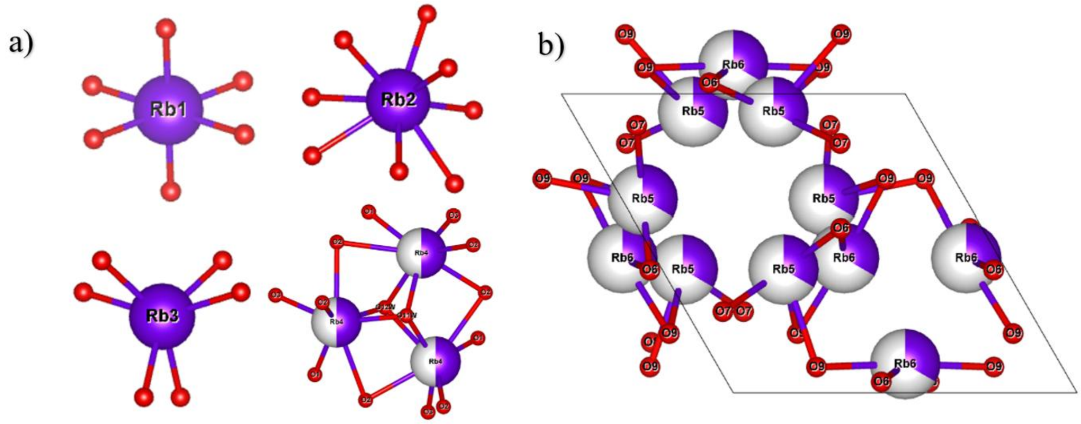

Figure S1. Rubidium coordination in **II**: (a) fully-occupied Rb(1), Rb(2), and Rb(3) sites and partially occupied Rb(4); (b) disordered and partially-occupied Rb(5) and Rb(6) sites.

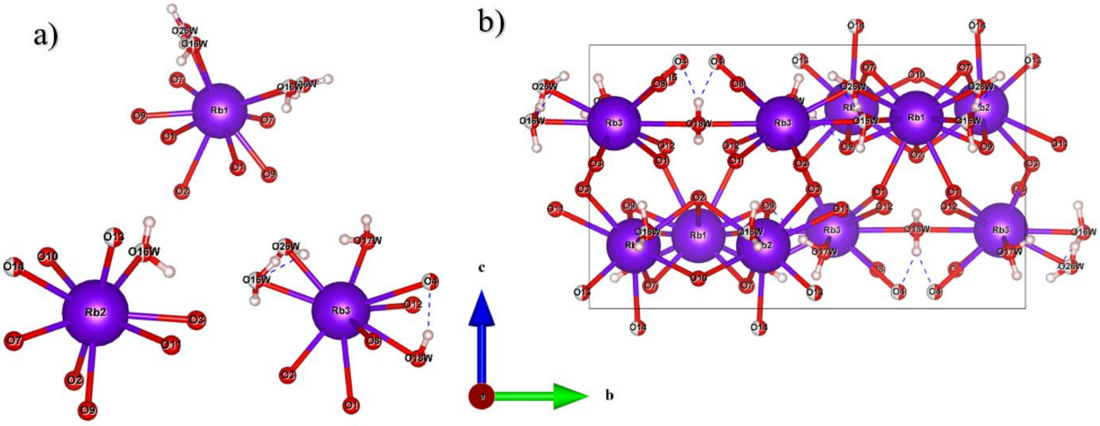

Figure S2. Rubidium coordination in **III**: (a) three unique Rb(1), Rb(2), and Rb(3) sites; (b) extended rubidium-oxygen bonding.

Table S1. Site occupancy of Rb vs Na obtained by free variable refinement of each unique alkali site in **IV**.

	Rb	Na
Site 1	0.905(3)	0.095(3)
Site 2	0.799(3)	0.201(3)
Site 3	0.785(3)	0.215(3)
Site 4	0.420(2)	0.580(2)
Site 5	0.298(3)	0.702(3)

Table S2. Hydrogen bonding interactions originating from iron-coordinated water molecules in compound **V**.

Compound	D-H···A	*d* (O-H) [Å]	*d* (H···O) [Å]	*d* (O···O) [Å]	∠ (O-H···O) [°]	Acceptor atom symmetry code
**V**	O31W-H···O13	0.88(2)	1.80(3)	2.647(6)	162(7)	x, -y+1, z+1/2
**V**	O31W-H···O17	0.89(2)	1.76(3)	2.648(6)	175(7)	-x+3/2, -y+3/2, -z+2
**V**	O32W-H···O18	0.88(2)	1.80(3)	2.676(7)	172(7)	-x+3/2, -y+3/2, -z+1
**V**	O32W-H···O23	0.88(2)	1.79(3)	2.667(7)	171(8)	-x+3/2, y+1/2, -z+3/2
**V**	O33W-H···O25	0.86(2)	1.88(3)	2.720(7)	163(7)	x, -y+1, z+1/2
**V**	O33W-H···O26	0.87(2)	1.85(3)	2.695(7)	168(8)	x, -y+1, z+1/2

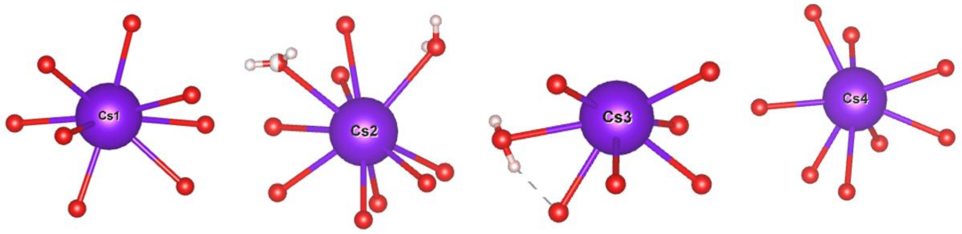

Figure S3. Cesium coordination of fully-occupied Cs sites in **V**.

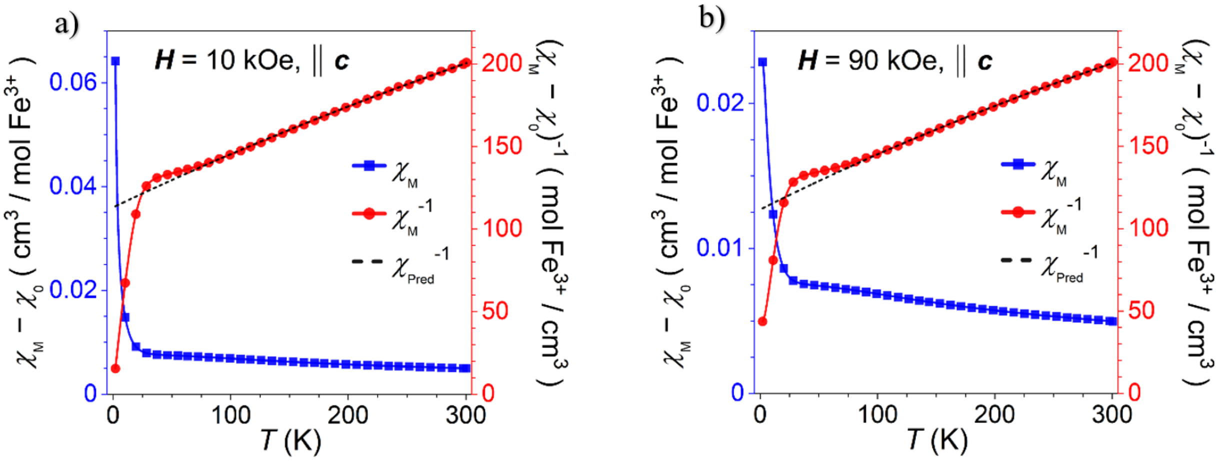

Figure S4. Curie-Weiss fitting curves for H = 10 kOe (a) and 90 kOe (b) with the field oriented parallel to the c-axis. The predicted fit lines are given and accentuate the antiferromagnetic deflections from the fit line in the 20-100 K range. These deflections are larger for H = 90 kOe and are attributed to the increase in spin torquing from the increasing applied field strength.

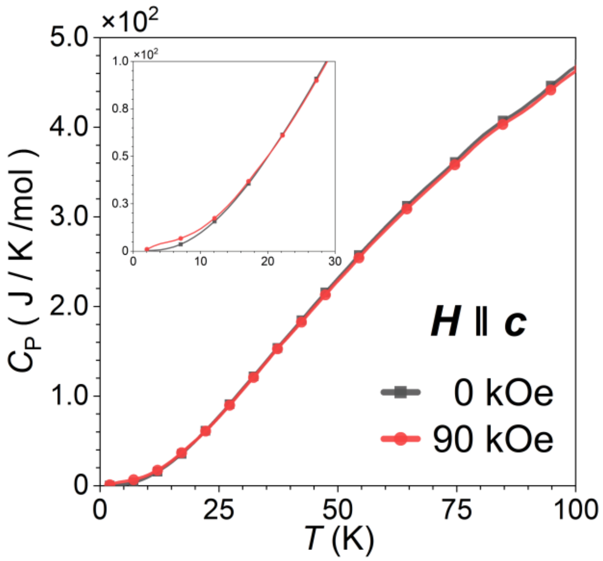

Figure S5. Heat capacity of 0.6 mg single crystal of **I** oriented so the applied field is parallel to the c-axis. Inset is of the subtle low-temperature deviation of 0 and 90 kOe applied fields, perhaps attributed to spin torquing reorientations at low temperatures as thermal energy if frozen out.
